# The effects of geometric uncertainties on computational modelling of knee biomechanics

**DOI:** 10.1098/rsos.170670

**Published:** 2017-08-09

**Authors:** Qingen Meng, John Fisher, Ruth Wilcox

**Affiliations:** Institute of Medical and Biological Engineering, School of Mechanical Engineering, University of Leeds, Leeds, UK

**Keywords:** the knee, biomechanics, cartilage, meniscus, geometric uncertainties, computational modelling

## Abstract

The geometry of the articular components of the knee is an important factor in predicting joint mechanics in computational models. There are a number of uncertainties in the definition of the geometry of cartilage and meniscus, and evaluating the effects of these uncertainties is fundamental to understanding the level of reliability of the models. In this study, the sensitivity of knee mechanics to geometric uncertainties was investigated by comparing polynomial-based and image-based knee models and varying the size of meniscus. The results suggested that the geometric uncertainties in cartilage and meniscus resulting from the resolution of MRI and the accuracy of segmentation caused considerable effects on the predicted knee mechanics. Moreover, even if the mathematical geometric descriptors can be very close to the imaged-based articular surfaces, the detailed contact pressure distribution produced by the mathematical geometric descriptors was not the same as that of the image-based model. However, the trends predicted by the models based on mathematical geometric descriptors were similar to those of the imaged-based models.

## 1. Introduction

Osteoarthritis (OA) is the most common joint disease, affecting an estimated 10% of the world's population over 60 [1]. While all diarthrodial joints can develop OA, it is more common in the knee than other joints [1]. Late stage total knee replacement surgery is relatively successful, but increasing patient activity levels and the rise in obesity are placing extra demands on joint replacements. This has led to increasing effort to develop earlier stage tissue-sparing interventions for knee OA to delay the need for replacement surgery.

To develop and evaluate devices for knee cartilage or meniscus repair requires an understanding of the mechanical environment within the joint. However, investigating the biomechanics of the knee is highly challenging, particularly *in vivo* where experimental approaches must largely be non-invasive. Computational models have played an important role in understanding knee mechanics, because they are non-invasive and can provide information that would be difficult or impossible to obtain from experimental and clinical studies. Such models have been used to study the role of the articular cartilage structure [2–4], and the effects of surface injury [5], osteochondral defects [6] and meniscectomy [7–9].

Most of the previous computational models of the knee have used the finite-element (FE) method due to its ability to represent the complex geometry. A number of factors affect the accuracy of the FE model predictions, including the geometry, material properties and boundary conditions. While the material properties of the meniscus and cartilage have been extensively studied [2–4,9], there has been limited evaluation of the effects of the representation of the geometry of the tissues.

Previous models of the natural knee have been generated either from simplified mathematical descriptors of the knee anatomy or directly from three-dimensional image data (referred to here as ‘image-based models’). In both cases, there will be a level of uncertainty in the accuracy of the geometric representation of the cartilage and meniscus.

Models based on mathematical descriptors have been widely used to represent the geometry of the knee in computational models [10–12]. The advantage of this approach is that the geometries of the tissues can be easily generated using software drawing tools, and the resulting shapes readily meshed for FE analysis. The time required to develop the model is generally lower than for image-based models. However, the level to which these models represent the real knee joint anatomy, and the resulting uncertainty in the predicted outputs, have not been well examined.

Image-based models, which are most commonly based on magnetic resonance imaging (MRI) [9,13–20], have the advantage that detailed geometric features can be captured. However, the accuracy of the geometry obtained from this method is limited by the resolution of the MRI and the accuracy of the segmentation technique. While sub-pixel accuracy in the segmentation has been reported [21], Van Den Broeck *et al*. [22] found the errors of the three-dimensional reconstruction from MR images can be as large as two pixels. It is common in MRI to use a slice thickness larger than the in-plane resolution [2,19,20]. Such an imaging scheme will add additional uncertainties in the direction of the slice thickness. Furthermore, because cartilage and meniscus are viscoelastic, further uncertainty is introduced by the level of pre-deformation of the tissues when the joint is scanned.

Some level of geometric uncertainty will therefore inevitably exist in both types of computational model of the knee. If the effects of these uncertainties are not evaluated, the level of reliability of the model predictions cannot be determined. However, to date there has been only limited work to examine these effects [23]. Therefore, the aim of this study was to evaluate the sensitivity of predicted knee contact mechanics to the geometric uncertainties in the representation of the cartilage and meniscus. Image-based and polynomial-based FE models of the knee were compared and sensitivity studies undertaken to evaluate the effects of the geometric uncertainties.

## 2. Models and materials

### 2.1. Overall study design

Image-based and polynomial-based FE models of the same condyle were used in this study. The two model types were initially compared without the meniscus component to isolate the effects of the cartilage representation from those of the meniscal conformity. Different orders of the polynomial function were also examined. Models with the meniscus were then used to undertake sensitivity studies to evaluate the effects of uncertainties due to image resolution and segmentation. The sensitivity studies were also used to compare the trends seen between model types.

### 2.2. Model geometry

**Figure 1.**
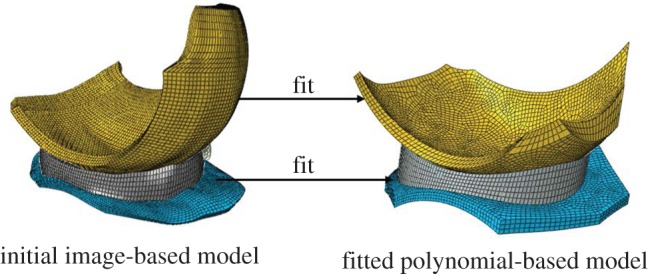
The initial image-based and polynomial-based (fourth order) models of one condyle with a meniscus used in this study.

The knee model developed by the Open Knee project [24,25] was adopted for the image-based models. This model had been segmented from the MR images of the right knee of a 70-year-old female, which were acquired with a resolution of 0.29 mm pixel^−1^ and a slice thickness of 1.5 mm. The imaging technique used a three-dimensional spoiled gradient echo sequence with fat suppression, TR = 30, TE = 8.9, Flip Angle = 35, Field of View = 150 mm × 150 mm [24]. To reduce confounding factors due to load distribution between the two condyles, only the medial condyle was considered (figure 1).

In the polynomial-based models, the femoral and tibial articular surfaces were represented by polynomial functions fitted to the image-based model (figure 1). Three polynomial models were investigated using 3rd (P3), 4th (P4) and 5th (P5) degree polynomial functions. The P3, P4 and P5 models were generated by fitting surfaces to the articular surfaces of the femoral and tibial cartilage of the image-based model using the *fit* function in MATLAB (Version R2013a, The MathWorks, Inc., USA). This function uses the least-squares method to fit surfaces to data and returns goodness-of-fit statistics such as the coefficient of determination and root mean squared error (RMSE). In the polynomial-based intact model, the meniscus was created so that the top and bottom surfaces perfectly conformed to the articular surfaces of the femoral and tibial cartilage, respectively (figure 1).

**Figure 2.**
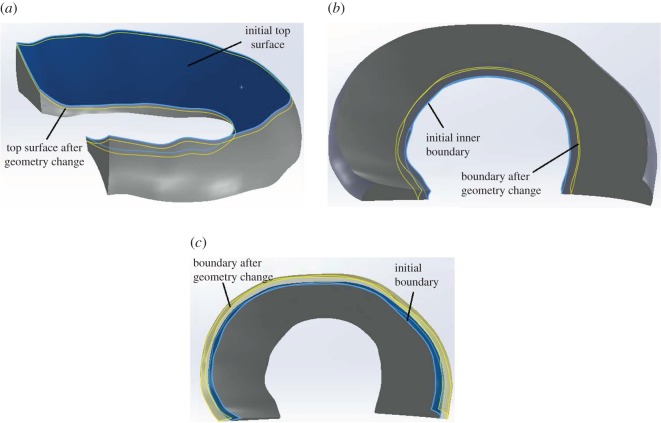
Schematic diagram of the method for reducing the height (*a*), increasing the inner boundary radius (*b*) and increasing the outer boundary radius (*c*) of the meniscus for the sensitivity study.

The effects of the geometric uncertainties caused by the limitations of the MRI resolution and accuracy of the segmentation were investigated by varying the meniscal geometry of the intact models (figure 2). As mentioned in the Introduction, the accuracy of segmentation may vary [21,22]. Therefore, a large geometric variation in the meniscus was investigated to cover a wide range of uncertainties. The investigated variations were −0.2 to 0.2 mm in the height and up to 1.0 mm in the inner and outer radius of the meniscus.

### 2.3. Materials

As the focus of this study was on the effects of geometric uncertainties, the biphasic mechanical behaviour [9,16,26,27] of the cartilage and meniscus were not considered in this study. Therefore, the cartilage was assumed as linear isotropic and meniscus transversely isotropic. The elastic modulus and Poisson's ratio of the cartilage were 15 MPa and 0.46 [20], respectively. The material properties of the meniscus were [20]: *E*_f_ = 120 MPa, *E*_p_ = 20 MPa, *ν*_p_ = 0.2, *ν*_fp_ = 0.3, *G*_fp_ = 57.7 MPa, *G*_p_ = 8.33 MPa, where the subscript ‘f’ denotes the fibre direction in the meniscus, ‘p’ denotes the plane orthogonal to the fibre direction.

### 2.4. Loading and boundary conditions

All models were solved at the full extension position and only the vertical loading was applied to the models to simulate two-legged stance. To investigate the differences between the image-based and polynomial-based models under a range of contact conditions, loads between 100 and 1500 N were applied to the models without a meniscus. The contact force of the tibiofemoral joint under two-legged stance is approximately equal to body weight [28]. However, because only one condyle of the joint was considered in this study, 400 N (approximately half body weight) was applied to the intact models.

In all models, the bottom surface of the tibial cartilage was fixed to simulate the interface between the tibial cartilage and tibia. The femoral cartilage was only allowed to move vertically to simulate two-legged stance. The top surface of the femoral cartilage (the interface between bone and cartilage) was constrained with a coupling constraint option to a reference point located at the centre of the condyle. The loading and boundary conditions of the femoral cartilage were applied to this reference point.

In the intact models, meniscal movements were restricted by linear spring elements [20,29], simulating the meniscal attachment ligaments. The end nodes of each meniscal horn were fixed to anchorage points of meniscal attachments, and the stiffness of each attachment (the sum of all spring elements at each horn) was 2000 N mm^−1^ [20,29].

### 2.5. Solutions and mesh

The FE models were solved using Abaqus (v. 6.13, Dassault Systems Simulia Corp, USA). Hexahedral elements were used for all the models. Mesh convergence studies were performed for all model types, until the change in predicted contact area caused by doubling the number of the elements was less than 3%. The resulting number of elements was approximately 68 000 and 46 000 for the image-based and polynomial-based intact models, respectively, and 25 000–30 000 for the models without meniscus.

## 3. Results

### 3.1. Comparison between polynomial-based and image-based models without meniscus

**Figure 3.**
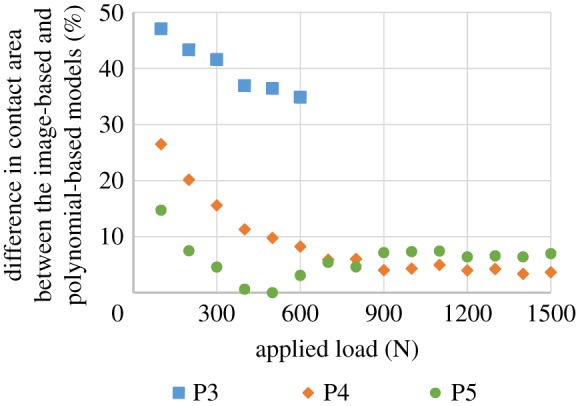
The relative difference in the predicted contact area between the image-based and polynomial-based models without a meniscus under different loads (for the loading cases larger than 600 N, the P3 model did not converge due to excessive compressive strain (larger than 30%)).

With the increase in the degree of the polynomial function, the fitted polynomial-based surfaces were found to approach the image-based surface, with RMSE of under 0.4 mm for the P5 case (table 1). A comparison of the outputs of the FE models is presented in table 2 and figure 3. Under lower loads (i.e. less than 600 N), the contact areas predicted by the higher degree polynomial-based models were always closer to those of the image-based model than the lower degree polynomial-based models. For the heavier loading cases (i.e. larger than 700 N), the P3 model did not converge due to excessive compressive strain (larger than 30%). Under heavier loading, the differences in the contact area between the P4 and P5 models and the corresponding image-based model were very small (less than 8%) and appeared to be relatively constant (figure 3). However, it should be noted that the contact pressure contours of all the polynomial-based models clearly differed from those of the image-based model (table 2).

**Table 1.** The coefficient of determination of the fitted polynomial surfaces with the initial image-based surfaces.

		3-degree	4-degree	5-degree
tibial cartilage	coefficient of determination	0.947	0.975	0.987
	RMSE (mm)	0.740	0.510	0.366
femoral cartilage	coefficient of determination	0.986	0.993	0.997
	RMSE (mm)	0.437	0.310	0.206

**Table 2.** Comparison of the contact behaviour between the image-based and three polynomial-based models without a meniscus.

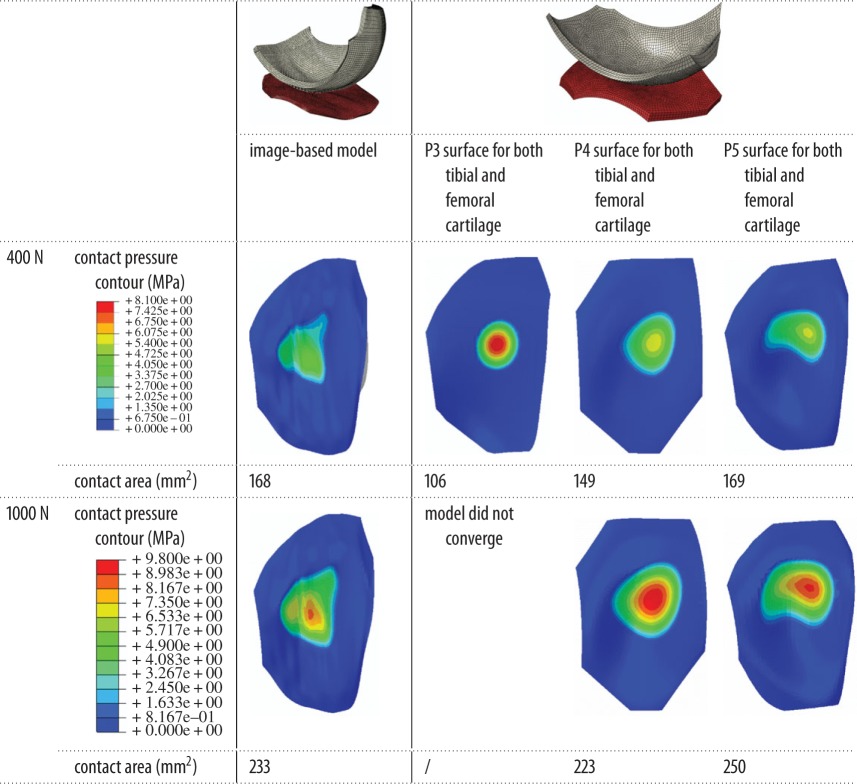

### 3.2. Sensitivity to meniscus geometry

**Figure 4.**
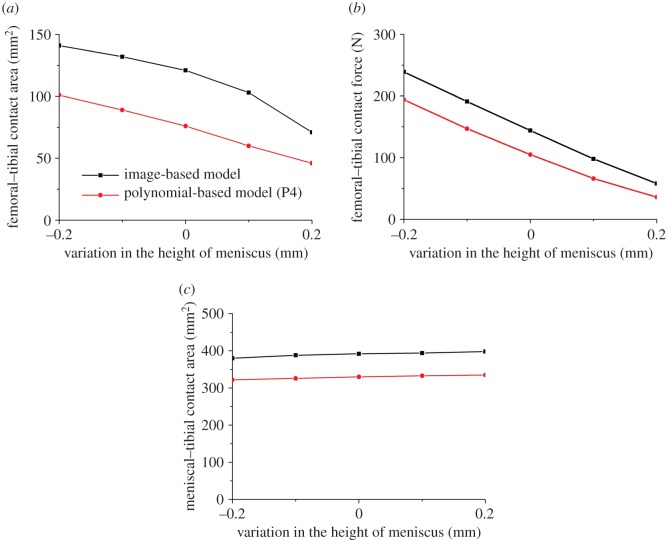
The sensitivity of the femoral–tibial contact area (*a*), femoral–tibial contact force (*b*) and meniscal–tibial contact area (*c*) to the small geometric variation in the height of meniscus.

With the intact model, a small reduction in the height of the meniscus caused considerable increases in the contact area and contact force at the femoral–tibial interface and slight decrease in the contact area at the meniscal–tibial interface (figure 4). In both polynomial-based (P4) and image-based models, increasing the height of the meniscus by 0.2 mm resulted in a 40% and 60% reduction in the contact area and contact force at the femoral–tibial interface, respectively, but less than 5% change in the contact area at the meniscal–tibial interface (figure 4).

**Figure 5.**
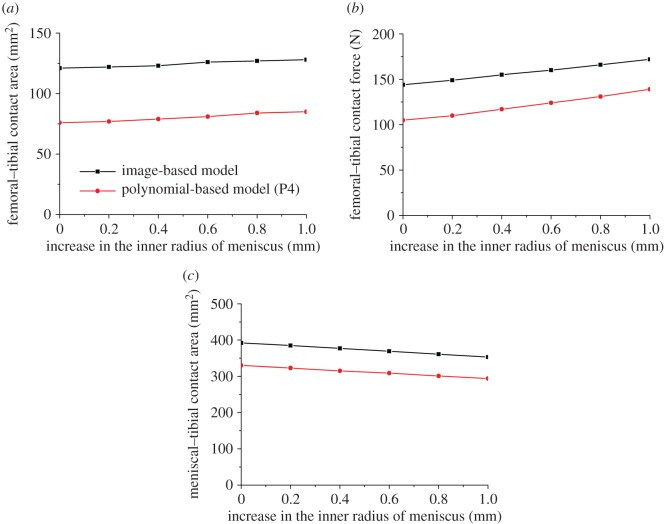
The sensitivity of the femoral–tibial contact area (*a*), femoral–tibial contact force (*b*) and meniscal–tibial contact area (*c*) to the small geometric variation in the inner radius of the meniscus.

**Figure 6.**
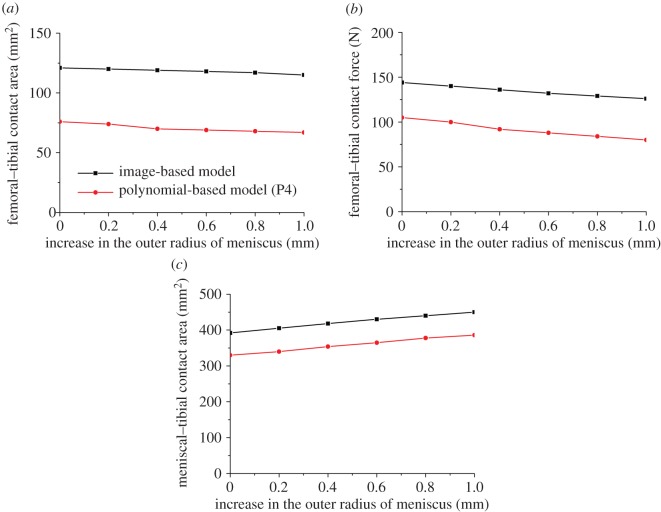
The sensitivity of the femoral–tibial contact area (*a*), femoral–tibial contact force (*b*) and meniscal–tibial contact area (*c*) to the small geometric variation in the outer radius of the meniscus.

The contact behaviour of the intact knee models was less sensitive to the small variations in the inner and outer radius of the meniscus than the height of the meniscus. With both image-based and polynomial-based (P4) models, the changes in the investigated outputs caused by increasing the inner radius of the meniscus by 0.2 mm were all less than 5% (figure 5). The 0.2 mm variation in the outer radius showed a similar trend (figure 6). Moreover, the changes in the contact behaviour of the intact joint caused by a much larger variation in the inner radius and outer radius (i.e. 1 mm) were still not as large as the 0.2 mm variation in the height. Increasing the inner radius of the meniscus by 1.0 mm in the image-based model resulted in 6% and 19% increase in the femoral–tibial contact area and contact force, respectively, and 10% reduction in the meniscal–tibial contact area (figure 5). A decrease of 5% and 13% and increase of 15% were caused by the 1.0 mm increase in the outer radius for the femoral–tibial contact area, femoral–tibial contact force, and meniscal–tibial contact area, respectively (figure 6).

### 3.3. Comparison between polynomial-based and image-based models with meniscus

Owing to the differences in conformity between the meniscus and the cartilage in the image-based and polynomial-based models, there was not an exact match in the initial conditions prior to the load application, so one-to-one comparisons of the model predictions were not undertaken. However, similar trends were seen for the image-based and polynomial-based (P4) models across all of the sensitivity studies. There was a high degree of correlation between the two models for changes in meniscal height (*R*^2^ > 0.97 for all outputs). For the effect of the inner radius of the meniscus, the correlation coefficients between the polynomial-based (P4) and image-based models for the femoral–tibial contact area, femoral–tibial contact force and meniscal–tibial contact area were 0.975, 0.999 and 0.999, respectively. The corresponding correlation coefficients for the effect of varying the outer radius of the meniscus were 0.967, 0.995 and 0.999, respectively.

## 4. Discussion

The geometry of the articular components of the knee is an important factor in predicting the joint contact mechanics. In current computational models of the knee, there are a number of factors that lead to uncertainty in the geometric representation, including the underlying resolution of MRI, the accuracy of the segmentation and the application of simpler geometric descriptors. Evaluating the effects of these geometric uncertainties is fundamental to understanding the level of the reliability of current and future computational knee models. Li *et al*. [23] found that user variability in processing MR images caused a thickness variation in the cartilage component that resulted in approximately a 10% change in peak contact stress. However, the effects due to the resolution of the MR images, the geometric uncertainties in the meniscus and the representation of the surfaces using mathematical descriptors are still not clear. The main aim of this study was to investigate these effects.

The comparison between the image-based and the polynomial-based models without a meniscus indicated that the fitted polynomial surfaces with higher degree were very close to the articular surface of the image-based model (table 1). Moreover, the contact areas predicted by the polynomial-based models can be very close to those of the image-based model (table 2 and figure 3). However, the detailed contact pressure distributions produced by the polynomial-based models were not the same as those of the image-based model (table 2). These observations are consistent with the outcomes seen in similar studies of the hip [30]. The differences in the contact pressure contours between the polynomial-based models and the image-based model may be caused by the irregularities on the cartilage surfaces of the image-based model. Such surface irregularities could not be reflected by the polynomial-based surfaces. The finding that the P4 and P5 models produced closer contact area to the image-based model under higher loads indicated that the effects of the surface irregularities had less of an influence when the contact area was relatively large. However, their effects on the contact pressure contour remained considerable in all cases (table 2).

The polynomial-based models developed in this study were much closer to the image-based model than many of the simpler geometrical models used in previous studies [10–12], so it is reasonable to assume that previous models would not produce the same contact pressure as corresponding image-based models. It should also be noted that whether the image-based model is a closer representation of the actual cartilage geometry than the polynomial-based model is still to be investigated. It could be argued that the image-based models may be more accurate because they reflect the local geometric characteristics. However, whether the local surface irregularities of the image-based models are real or artefacts caused by the resolution of MRI needs to be confirmed by more accurate image-based models and experimental tests.

The comparison of the contact pressure between the image-based and polynomial-based models also has important implications for understanding the effects of the geometric uncertainties of current image-based computational knee models. Most of the current MRI-based FE knee models use a resolution between 0.2 and 0.6 mm [2,9,20]. It has been reported that the errors in the three-dimensional reconstruction from MR images varies from sub-pixel to two pixels [21,22]. Therefore, the errors in the cartilage geometry of the FE knee models resulting from the MRI scanning and segmentation can be easily larger than 0.2 mm, which are at the same magnitude as the RMSE of the P5 image-based model (0.21–0.36 mm). Thus, the uncertainties in cartilage surfaces caused by the resolution of MRI and accuracy in segmentation may have an effect of the same magnitude as the RMSE of the P5 image-based model.

The effects of the geometric uncertainties caused by the resolution of the MRI and accuracy in segmentation were confirmed by the sensitivity study where the effects of small variations in the meniscal size of the intact models were investigated. As predicted by both polynomial-based and image-based models, increasing the height of the meniscus by only 0.2 mm, which is at the same magnitude as the potential geometric errors resulting from the MRI scanning and segmentation, caused a considerable reduction in the contact area and contact force at the femoral–tibial interface (40% and 60%, respectively).

The prediction of knee contact mechanics was less sensitive to the small variations in the inner and outer radius of the meniscus than the height of the meniscus. This finding has an important implication for reducing the uncertainties caused by the resolution of the MR images when image-based computational knee models are created. The height of the meniscus should always be captured by the highest resolution in the settings. For example, when a knee joint is scanned with a higher in-plane resolution (e.g. 0.2 mm) and a large slice thicknesses (e.g. 1.5 mm), it should be scanned in the sagittal or coronal plane rather than in the transverse plane. Moreover, because cartilage and meniscus exhibit viscoelastic characteristics, if it is compressed under loading before scanning, it may take hundreds of seconds to return to its natural non-weightbearing position. Therefore, the initial loading conditions of the joint need careful consideration before imaging.

The comparison of the contact mechanics between the image-based and polynomial-based models with the meniscus also indicated the reliability of previous models. Indeed, for all the mechanical outputs investigated in this study, the trends predicted by the fitted polynomial models were similar to those of the image-based model with strong linear correlations (figures 5 and 6). Therefore, even if there are quantitative differences between the results predicted by the image-based and polynomial-based models, it is reasonable to believe that the qualitative conclusions predicted by the previous models using simpler mathematical geometrical descriptors should be reliable.

There are some limitations in this study. First, only one loading condition was considered representing two-legged stance. The effects of geometric uncertainties under other loading conditions should be considered in the future in order to evaluate the full range of articular tissue in contact. Second, this study was based on only one subject. Whether the same conclusions would be obtained across different subjects with varying knee anatomy is another important issue to be addressed in future studies. Furthermore, the results in this study were not directly validated by experiments and the reliability of the image-based models was not evaluated. As a result, as discussed above, it is not possible to determine unequivocally whether the image-based or polynomial-based models better represent the true physiological conditions from this study.

Despite these limitations, this study suggests that the geometric uncertainties in cartilage and meniscus resulting from the resolution of MRI and accuracy of segmentation may cause considerable uncertainty in the predicted knee mechanics. Moreover, even if the mathematical geometric descriptors can be very close to the articular surface of the image-based model, the detailed contact pressure produced by the polynomial-based models is not the same as that of the image-based model. However, the models based on mathematical geometric descriptors can predict qualitatively reliable trends. These conclusions provide new insights into understanding the reliability of current and future computational models of the knee, which in turn are important for the development and pre-clinical evaluation of cartilage and meniscus repair interventions.

## Data accessibility

The data associated with this paper are openly available from the University of Leeds Data Repository (http://archive.researchdata.leeds.ac.uk/168/) [31].
